# Effects of Fe^2+^/Fe^3+^ Binding to Human Frataxin and Its D122Y Variant, as Revealed by Site-Directed Spin Labeling (SDSL) EPR Complemented by Fluorescence and Circular Dichroism Spectroscopies

**DOI:** 10.3390/ijms21249619

**Published:** 2020-12-17

**Authors:** Davide Doni, Leonardo Passerini, Gérard Audran, Sylvain R. A. Marque, Marvin Schulz, Javier Santos, Paola Costantini, Marco Bortolus, Donatella Carbonera

**Affiliations:** 1 Department of Biology, University of Padova, Viale G. Colombo 3, 35131 Padova, Italy; davide.doni.2@phd.unipd.it (D.D.); paola.costantini@unipd.it (P.C.); 2 Department of Chemical Sciences, University of Padova, Via F. Marzolo 1, 35131 Padova, Italy; leonardo.passerini@studenti.unipd.it (L.P.); donatella.carbonera@unipd.it (D.C.); 3 Institut de Chimie Radicalaire, Aix Marseille Universitè, CNRS, ICR, UMR 7273, Case 551, Ave Escadrille Normandie Niemen, CEDEX 20, 13397 Marseille, France; g.audran@univ-amu.fr (G.A.); Sylvain.marque@univ-amu.fr (S.R.A.M.); marvin.schulz@univ-amu.fr (M.S.); 4 Departamento de Química Biológica, Instituto de Biociencias, Biotecnología y Biomedicina (iB3-UBA), Facultad de Ciencia Exactas y Naturales, Universidad de Buenos Aires, Intendente Güiraldes 2160—Ciudad Universitaria, 1428EGA CONICET, Godoy Cruz 2290, Buenos Aires C1425FQB, Argentina; javiersantosw@gmail.com; 5 Instituto de Química y Fisicoquímica Biológicas Dr. Alejandro Paladini, Universidad de Buenos Aires, CONICET, Junín 956, Buenos Aires 1113AAD, Argentina

**Keywords:** frataxin, iron, EPR, fluorescence, CD, Fe-S cluster assembly machinery

## Abstract

Frataxin is a highly conserved protein whose deficiency results in the neurodegenerative disease Friederich’s ataxia. Frataxin’s actual physiological function has been debated for a long time without reaching a general agreement; however, it is commonly accepted that the protein is involved in the biosynthetic iron-sulphur cluster (ISC) machinery, and several authors have pointed out that it also participates in iron homeostasis. In this work, we use site-directed spin labeling coupled to electron paramagnetic resonance (SDSL EPR) to add new information on the effects of ferric and ferrous iron binding on the properties of human frataxin *in vitro*. Using SDSL EPR and relating the results to fluorescence experiments commonly performed to study iron binding to FXN, we produced evidence that ferric iron causes reversible aggregation without preferred interfaces in a concentration-dependent fashion, starting at relatively low concentrations (micromolar range), whereas ferrous iron binds without inducing aggregation. Moreover, our experiments show that the ferrous binding does not lead to changes of protein conformation. The data reported in this study reveal that the currently reported binding stoichiometries should be taken with caution. The use of a spin label resistant to reduction, as well as the comparison of the binding effect of Fe^2+^ in wild type and in the pathological D122Y variant of frataxin, allowed us to characterize the Fe^2+^ binding properties of different protein sites and highlight the effect of the D122Y substitution on the surrounding residues. We suggest that both Fe^2+^ and Fe^3+^ might play a relevant role in the context of the proposed FXN physiological functions.

## 1. Introduction

Frataxin (FXN) is a small acidic protein that is highly conserved in most organisms, from bacterial to mammalian. A low FXN expression, primarily caused by an abnormal GAA triplet repeat expansion in the first intron of the frataxin gene, is associated with the neurodegenerative disease Friedreich’s ataxia (FRDA; OMIM 229300) [1]; in addition, several FXN point mutations including nonsense, missense, insertions and deletions have been associated with compound heterozygous FRDA patients [1,2,3,4]. The main biochemical feature of FRDA is a large depletion of proteins relying on iron-sulphur clusters (ISC) for function (such as those of the mitochondrial respiratory chain complexes I, II and III or aconitase) with an accompanying increase in mitochondrial iron and oxidative stress [5,6,7,8].

Human FXN is nuclear encoded, expressed in the cytoplasm as a precursor of 210 amino acids, and then imported into the mitochondria, where it undergoes two-step maturation by the mitochondrial processing peptidase (MMP) [9,10,11]. MMP first cleaves a portion of the N-terminus, the mitochondrial import signal, originating an intermediate form (FXN 42–210), and with a further cleavage MMP produces the mature FXN 81–210, which is the most abundant form found both in normal individuals and in FRDA patients [9]. Sequence alignment studies have distinguished the N-terminal frataxin region, which is intrinsically unfolded and poorly conserved among the different species, from the C-terminus highly conserved block of about 100–120 amino acids, which is considered the most important part for protein function [12]. The 3D structure of human FXN has been determined by both X-ray crystallography [13] and NMR [14], and the agreement between the results suggests the high stability and rigidity of the structure. The structure of the conserved C-terminus (81–210) consists of a mixed alpha-beta fold with two helices packing against a contiguous anti-parallel beta sheet, assembled in the sequence alpha-(beta)_7_-alpha, which, despite its simplicity, is very rare. The N-terminal tail of human FXN (residues 81–92) was shown to be intrinsically unfolded [14]. Despite harboring a potential iron binding site [15], it is often truncated (obtaining FXN 90–210) for *in vitro* studies. 

The proposed role of FXN as an allosteric regulator of the biosynthetic iron-sulphur cluster (ISC) machinery has recently found further confirmations [16,17,18,19]. The ISC biosynthetic pathway takes place in the mitochondria and is a multi-step process involving several proteins [19,20,21]: the early cluster assembly requires a yet-unidentified iron source and is performed by a pentameric quaternary protein complex. The complex is formed by FXN, the cysteine desulfurase NFS1 (extracting the sulphide from free cysteine), ISD11 (an accessory protein), ACP (an acyl-carrier protein), and ISCU (the scaffold protein on which the clusters are assembled). The structure of this complex has been recently solved via cryo-electron microscopy, showing FXN to be at the interface between NFS1 and ISCU [16]. Additionally, given its well-known ability to bind ferrous and ferric ions, FXN has been proposed as the iron donor ISC machinery complex [22,23,24]. Finally, FXN has been proposed as a key regulator of iron homeostasis [25] and ferroptosis [26,27], a recently identified iron-dependent form of cell death [28]. 

The latter functions point to the critical importance of the interaction between iron ions and FXN. While the binding of ferrous/ferric iron is ascertained through a wide range of chemical and biological techniques, some incongruities among the collected data remain. Using fluorescence analysis and calorimetry titrations with both ferric and ferrous iron, it was found that human frataxin 81–210 binds 6–7 ferrous/ferric ions [22]. Differently, through a combination of fluorescence, NMR, and mass spectrometry, a binding stoichiometry of only three equivalents was reported for Fe^2+/3+^ and Co^2+^ ions [15]. An NMR study concluded that FXN binds only one Fe^2+^ equivalent and has no binding interaction with Fe^3+^ [24]. In a previous paper, using EPR and NMR spectroscopies, we showed that FXN is able to bind both iron forms [29]. Another contested feature of FXN is the tendency to aggregate in presence of ferrous or ferric ions. Mature human FXN 81–210, and also yeast and bacterial orthologs (Yfh1 and CyaY), do not aggregate *in vitro* when overexpressed and purified. However, only human FXN has been reported to retain the monomeric form when added with ferrous ions, while the orthologs show a clear tendency to aggregation [30,31]. While human FXN aggregation in the presence of ferric ions has been alternatively dismissed [32] or confirmed [33], Yfh1 and CyaY are prone to aggregation [34]. Since aggregation may affect the iron binding properties, it is important to determine its threshold in terms of protein concentration and compare it to the FXN concentrations commonly employed for the experimental determination of iron affinity. 

**Scheme 1:**
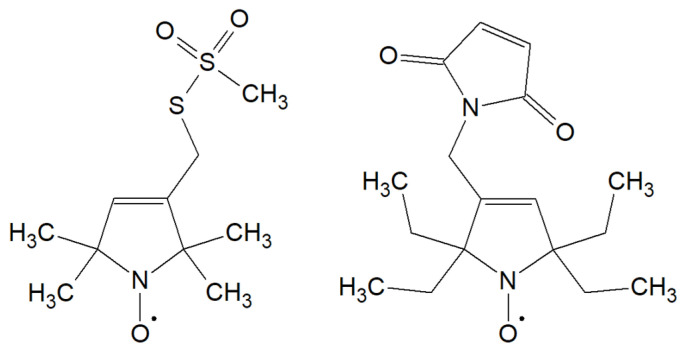
The MTSSL and the M-TETPO nitroxide spin labels.

In this work, we focus on the effects of ferrous/ferric iron binding on possible conformational changes of the mature human FXN 90–210 and its D122Y variant, the only pathological variant identified so far in the iron binding region. The effects of iron were explored also as a function of pH and protein concentration. We make use, for the first time, of the site-directed spin labeling (SDSL) technique coupled with electron paramagnetic resonance (EPR). This is a powerful tool for detecting changes in structure, dynamics, and oligomerization in proteins and was used here to get information on the protein structural changes following iron binding. We adopted two different spin labels, MTSSL and the reduction-resistant M-TETPO (see Scheme 1), to work with Fe^3+^ and Fe^2+^, respectively. The EPR investigation has been supplemented by tryptophan fluorescence quenching and circular dichroism measurements to allow also comparison with previously adopted methods. 

## 2. Results

### 2.1. Structural Remarks and Choice of Labeling Sites

**Figure 1:**
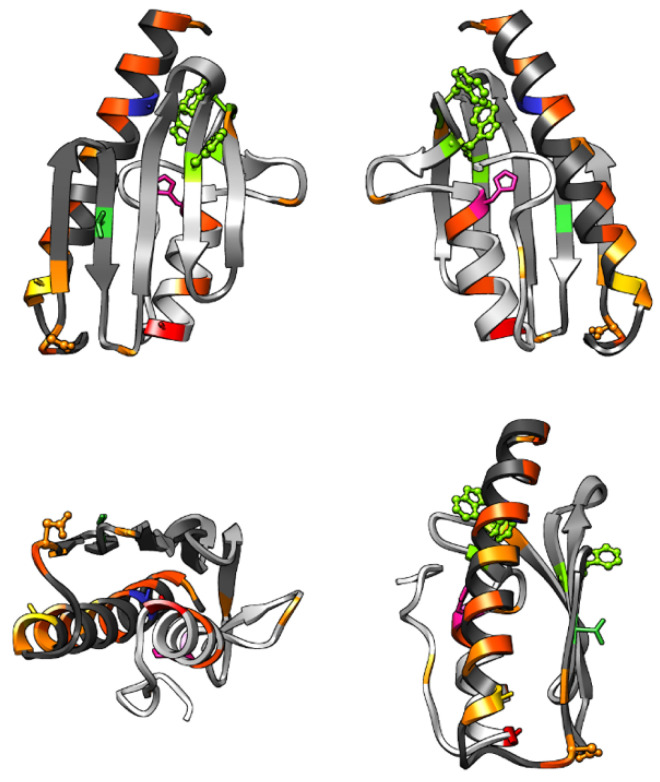
Structure of human FXN (PDB.ID 1EKG) from different angles, the N-terminus is in dark grey, the C-terminus is white. The Glu and Asp residues are colored dark and light orange, respectively. The three native Trp residues are colored bright green and shown in ball and stick representation. The spin labeling sites—A99 (blue), A114 (yellow), T133 (green), H183 (pink), A193 (red)—are shown in stick representation. The D122 residue in light orange, the site of the pathological D122Y mutation, is shown in ball and stick representation.

The physiological role of human FXN is still uncertain, as mentioned in the introduction, but its ability to bind iron in its ferrous and ferric oxidation states, along with other ions, seems to be a key part of its function. The iron binding region of WT human FXN and that of its pathological variants has been studied previously [29,35]: FXN uses Asp and Glu residues to bind iron via chelation by carboxylate groups. All of the long alpha-helix, located towards the N-terminal portion of the mature protein, as well as the following loop region, are rich in negatively charged residues and constitute the main iron-binding region (the positions of these residues are shown in orange in Figure 1). D122Y is the only pathological variant currently known that carries a mutation in the iron-binding region (shown in yellow in Figure 1); this mutation has been shown to affect the conformation of the loop region and to influence iron binding [29].

The spectroscopic study of human FXN reported so far have taken advantage of the three native Trp residues (W155, W168, W173) for fluorescence experiments; the Trp are shown in green in ball-and-stick representation in Figure 1. The W155 residue is in the beta-sheet region, exposed to the solution: this Trp is involved in the interface of the FXN/ISCU/NFS1 complex [16], and the W155R variant is one of the known pathological mutations [2]. The other two Trp are spatially close together, W168 and W173 are separated by about 0.6 nm, while W155 is more distant (1.4/1.7 nm to W168/172 respectively); W168 is partially exposed to the solution, while W173 is buried. The fluorescence probably comes from one or two of them since they are very close to each other and homo-FRET can easily occur. 

FXN has no native cysteine residues, and thus for SDSL EPR studies, cysteine mutants must be designed and expressed to place the spin label in the desired position. Five protein sites (namely A99, A114, T133, H183, A193, see Figure 1) were chosen with the aim of mapping possible changes in the protein structure and dynamics upon ferrous/ferric iron addition, probing the response of different protein regions. The A99 site is a buried site located at the beginning of the long alpha-helix, close to the iron binding region; A114, which is close to iron-binding sites and located to the end of the long alpha-helix, has been expressed both in the WT and in the D122Y variant; T133 belongs to the beta-sheet region involved in the interaction with the complex with ISCU/NFS1 [16]; H183 is located at the beginning of the second alpha helix, not involved either in the iron binding or the complex with ISCU/NFS1; A193 is located at the end of the second alpha helix, spatially close to the iron binding region. These sites have been singly mutated to Cys for the subsequent coupling reaction with nitroxide spin probes. Please note that the A99 position has a very limited solvent accessibility; therefore, it showed limited labeling efficiency (about 10%) and was thus viable only for the analysis with Fe^2+^ that was performed at higher concentration.

Since FXN is a small protein (MW = 14 kDa), its reorientation in aqueous solutions, at room temperature, is very rapid. This motion would mask any local contribution to the EPR spectral lineshape proper of the spin label; therefore, it is necessary to slow down the protein tumbling (quantified by the rotational correlation time, τ_c_) performing the EPR experiments in viscous solution. Ficoll PM70, a synthetic glucose polymer, at a 28% *w*/*w* concentration, was suggested as one of the better thickening agents [36]. We calculated the FXN rotational correlation time τ_c_, on the basis of the protein structure, at the viscosity of a buffer solution (η_Water_ = 8.94 × 10^−4^ Pa × s) and at the viscosity of a 28% *w*/*w* Ficoll solution (η_Ficoll_ = 1.92 × 10^−2^ Pa × s) using the program by M. Zerbetto et al. [37]: in buffer, τ_cWater_ was 5.6 ns, which is on a timescale comparable with that of the motions of the spin label side chain; in Ficoll, τ_cFicoll_ was 122 ns, slow enough to observe an EPR spectral shape influenced exclusively by the spin probe and backbone mobility, rather than the protein tumbling. 

### 2.2. EPR Spectra of FXN Interacting with Fe^3+^

**Figure 2:**
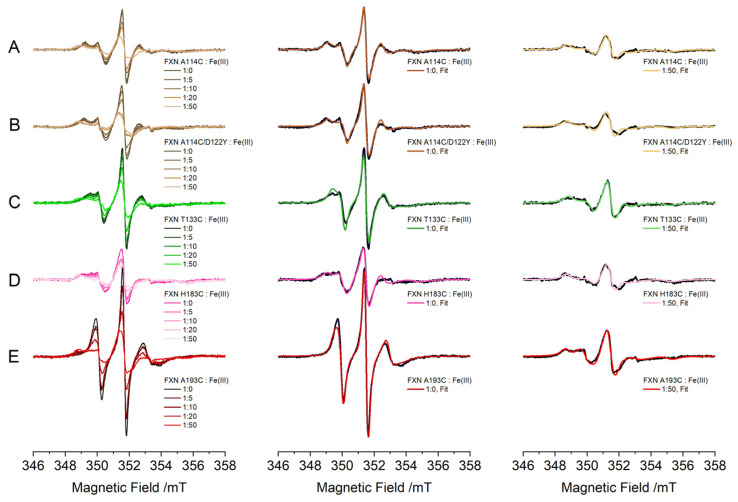
EPR spectra of FXN mutants at increasing protein: Fe^3+^ molar ratios from 1:0 to 1:50; protein concentration 10 µM, MTSSL label. The colors are the same of the positions highlighted in Figure 1: (**A**) A114C—brown; (**B**) A114C/D122Y—brown; (**C**) T133C—green; (**D**) H183C—pink; (**E**) A193C—red. Central and Right panels: EPR spectra (black) and simulations (color) of FXN mutants at protein: Fe^3+^ molar ratios 1:0 (central) and 1:50 (right).

The EPR spectra of FXN mutants in a 28% *w*/*w* Ficoll solution, were recorded at increasing protein: Fe^3+^ molar ratios at different FXN concentrations to explore both the effects of iron binding and the influence of protein concentration on the binding equilibrium. The spectra of all mutants at 10 µM concentration, labeled with MTSSL, are reported in Figure 2. As previously noted, mutant A99C showed very limited labeling efficiency (about 10%), which made it impossible to record EPR spectra at 10 µM concentration. 

In the absence of iron, each mutant shows a characteristic spin label mobility proper of the site, which confirms that the protein molecular tumbling has been slowed enough by the Ficoll solution; this allows spectral differences to be distinguished due to the specific local motion of the spin probe. Among these mutants, A193C showed the least restrained mobility, suggesting that WT FXN secondary structure is not very rigid at the edge of its second alpha helix. On the contrary, A114C, which is in a similar position at the end of FXN long alpha helix, has a more restrained mobility indicating a stable helical terminal. The other two labeled sites show a mobility that is intermediate between those two. In the A114C-D122Y mutant, MTSSL shows minimal differences in the spectra compared to the WT form, whereas the M-TETPO label shows some difference, the WT protein being more rigid than the pathological mutant in this region (*vide infra*). 

Upon addition of increasing amounts of Fe^3+^, all the labeled positions show a progressively restrained mobility both for the WT and the D122Y variant. In fact, the spectral broadening proceeds until an almost complete spin label immobilization is observed, at all labeled sites, as shown by the spectra at a FXN:Fe^3+^ 1:50 ratio in Figure 2. The onset of the immobilization, at a protein concentration of 10 µM, can be observed after the addition of about five equivalents of ferric iron. The rotational correlation time determined from the simulations of the spectra increases more than 2-fold going from a 1:0 to a 1:50 ratio, and, concurrently, the order parameter increases for all positions as shown in Table 1: this indicates that the motion of the labeled sites becomes slower and more constricted, consistent with the formation of a new interfacial contact hindering the probe mobility. 

**Figure 3:**
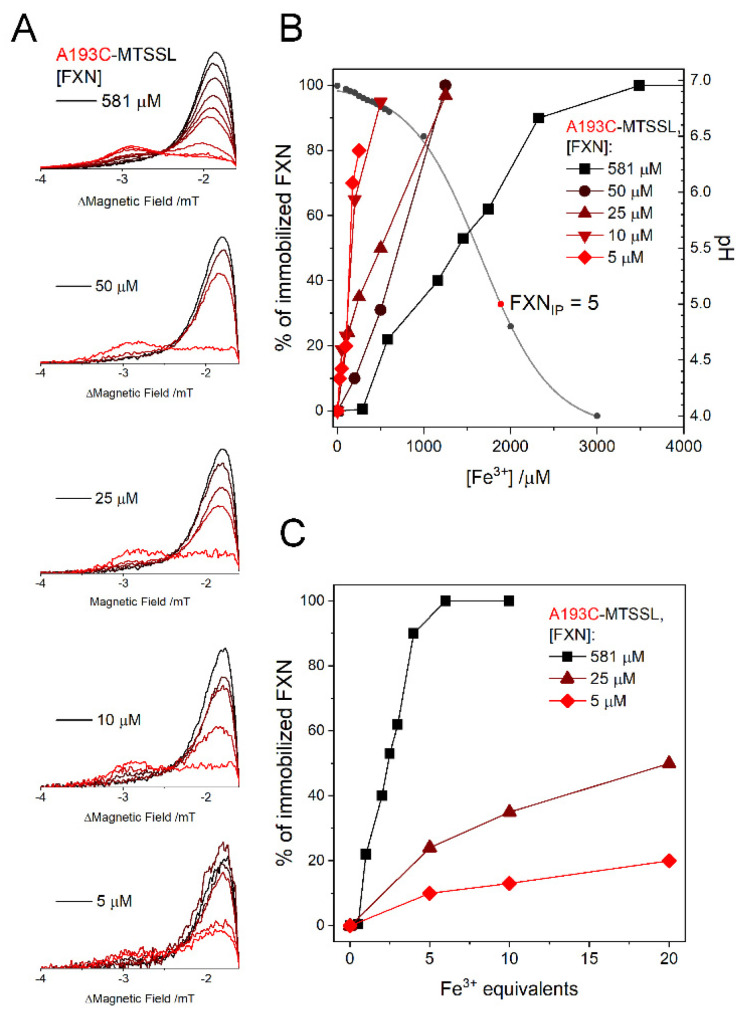
(**A**) From top to bottom, the left shoulder of the EPR spectrum of the A193C mutant labeled with MTSSL at progressively lower protein concentrations, the lighter the color the higher the FXN: Fe^3+^ ratio. (**B**) The percentage of immobilized protein (left axis, big dots) or the pH (right axis, small dots) vs. the nominal ferric iron concentration for different protein concentrations. (**C**) The percentage of immobilized protein vs. the FXN: Fe^3+^ molar ratio for three protein concentrations.

The immobilization of the protein depends not only on the protein:iron ratio, but also on the absolute protein concentrations. The dependence of the immobilization on protein concentration and pH has been explored using the A193C mutant that has the highest sensitivity to immobilization since it is the one that changes its mobility most dramatically; the zoom of the left shoulders of the EPR spectra are shown in Figure 3A. We must note that ferric iron at neutral pH is not present in solution as a free isolated ion, and the main species are colloidal oxo-hydroxo suspensions. The formation of these complexes upon addition of Fe^3+^ stock solution has a very strong acidifying effect on the sample solution, and even small additions of Fe^3+^ lead to a drop in pH. Therefore, we checked the effective pH as a function of Fe^3+^ concentration as shown in Figure 3B, right axis: under our buffering conditions, nominal Fe^3+^ concentrations of 1 mM and above steadily drop the pH below 6.5. Since FXN isoelectric point is IP = 4.9 [38], Fe^3+^ concentrations of 1.5 mM and above will cause protein aggregation by acidic denaturation rather than by a specific iron effect. Please note that EPR, fluorescence and CD experiments detailed in the text are all performed at pH ≥ 6.8.

The gradual spectral changes may be analyzed and reconstructed as combination of the two “pure” contributions (an example of the analysis is reported in the Supporting Information, Figure S1): (1) the initial, more mobile, nitroxide lineshape and (2) the final immobilized one, which does not change upon further Fe^3+^ addition. Importantly, the spectral analysis suggests that there are no further intermediate conformational states at significant concentration. The obtained percentage of immobilized protein (Figure 3B, left axis) has been plotted as a function of ferric iron nominal concentration concurrently with the effective pH of the solution (Figure 3B, right axis). Please note that irrespective of the final pH value, the immobilized shape is always the same, suggesting that high amounts of ferric iron lead to a denatured and/or strongly aggregated protein. 

The immobilization starts at progressively lower FXN:Fe^3+^ ratios as the protein concentration gets higher, as shown by the plot in Figure 3C. At the lowest concentration that we explored, 5 µM, the immobilization starts at 25 µM Fe^3+^, but the spectra are only partially immobilized (< 20%) even at a 1:20 FXN:Fe^3+^ molar ratio. 

Detection of the EPR spectra in buffered aqueous solution without the addition of Ficoll was also performed in labeled FXN. The EPR spectra were detected both in the absence of iron and in the presence of Fe^3+^. In the absence of iron, the spectra show three sharp peaks in agreement with the averaging effect on the spectra exerted by the protein tumbling due to the small size of the protein. Upon addition of Fe^3+^, the spectral broadening occurs as it was observed for the samples in Ficoll; the effect, however, is much less noticeable since the intensity is dominated by the sharper mobile component (see Figure S2 in the Supporing Information). Centrifugation of the sample, followed by EPR analysis of the supernatant, showed that the protein fraction that is immobilized precipitates along the colloidal iron, leaving behind the fraction that showed no motional broadening (see Figure S2). These results confirm the suggestion that Fe^3+^ addition promotes protein oligomerization [33], slowing down the molecular tumbling due to the bigger size of the Fe^3+^-FXN formed complexes. Analogous results were obtained in centrifugation experiments performed in Ficoll solutions (not shown); however, they are less clear cut, since a density gradient is formed.

The reversibility of the immobilization effect has been checked by adding EDTA in a three-fold excess relative to Fe^3+^ to remove the bound iron ions. After a one-hour incubation, the amount of immobilized component decreases (see the Supporting Information, Figure S3), suggesting that the aggregation is at least partially reversible.

### 2.3. EPR Spectra of FXN Interacting with Fe^2+^

EPR spectra have been collected on the different FXN mutants after addition of increasing amounts of Fe^2+^ to the samples, to investigate possible conformational changes induced by the binding of the divalent ion, which could be relevant for protein function. To prevent iron oxidation, the proteins have been prepared in strictly anaerobic conditions from the labeling stage and samples capillaries were prepared in an anaerobic glove box and sealed with wax at both ends. 

We observed a very rapid signal loss in FXN labeled with MTSSL following the addition of Fe^2+^. The reason for this lies in the quick reduction of the MTSSL nitroxide function to the EPR-silent nitroxylamine with concurrent oxidation of Fe^2+^ to Fe^3+^: this unwanted reaction not only results in signal loss, but also makes it impossible to separate the spectral changes induced by Fe^2+^ from those induced by Fe^3+^. For this reason, we changed the spin label to M-TETPO, a non-commercial spin label designed to be resistant to the cellular reducing environment [39]. Indeed, we verified that M-TETPO is also resistant to the reduction by Fe^2+^ in solution, making it the ideal probe in EPR studies of Fe^2+^ binding. We also confirmed that the M-TETPO-labeled protein shows the same behavior with Fe^3+^ as the MTSSL-labeled one (data reported in the Supporting Information, Figure S4).

**Figure 4:**
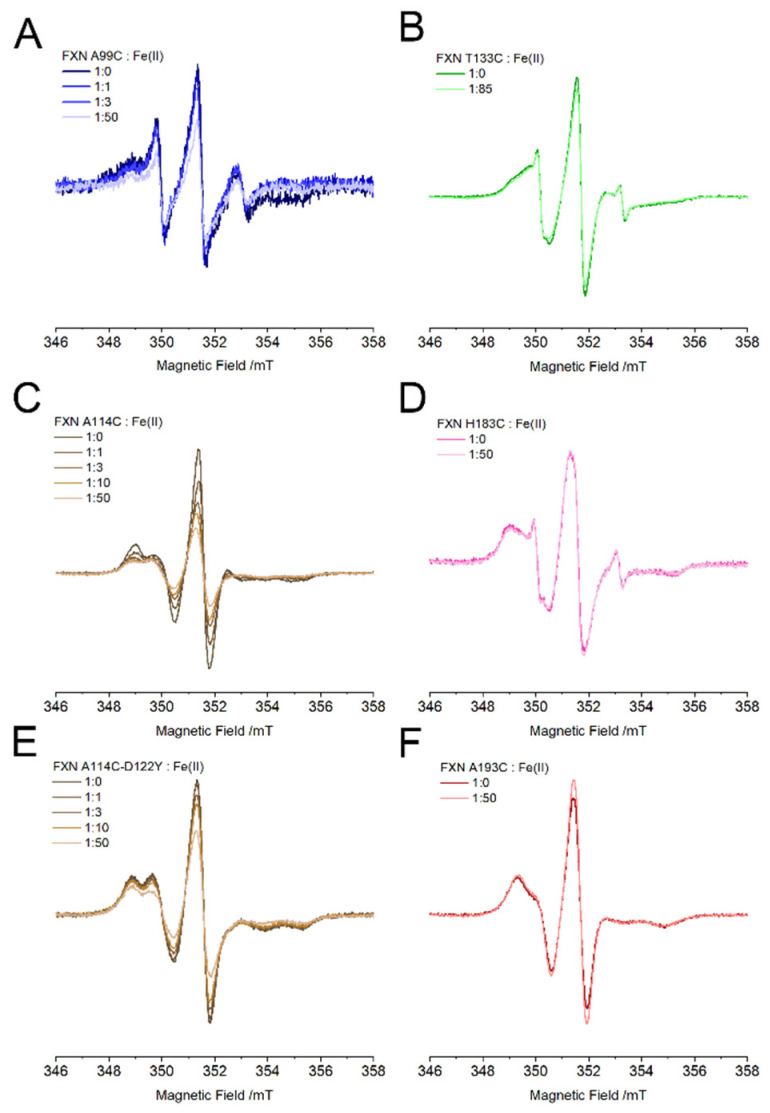
EPR spectra of different FXN mutants at increasing protein: Fe^2+^ molar ratios from 1:0 to 1:50; protein concentration 50 µM, M-TETPO label. (**A**) A99C—blue; (**B**) T133C—green; (**C**) A114C—brown; (**D**) H183C—pink; (**E**) A114C-D122Y brown; (**F**) A193C—red.

The EPR spectra of FXN mutants labeled with M-TETPO in the presence of ferrous iron are reported in Figure 4 (samples in Ficoll, protein concentration 50 µM), the spectra recorded at 10 µM protein concentration were identical to the ones reported here save for the worse S/N ratio. When iron is not present, the spectra show a reduced mobility compared to FXN labeled with MTSSL, indicating that the side chain of M-TETPO has less degrees of freedom than the one of MTSSL. As mentioned above, unlike MTSSL, M-TETPO differentiates the A114C-D122Y mutant from the WT counterpart, the spectra of the former revealing a faster mobility which indicates a change in the local structure of the D122Y FXN around residue 114. The difference between the labels is determined by the different conformers and mobility of their sidechain.

Once ferrous iron is added to the solution, we observe that the spectra of the sites that are located far from the iron binding region are unchanged even at a 1:50 FXN: Fe^2+^ ratio (T133, H183, A193, Figure 4B,D,F), while those close to the iron binding sites show an EPR intensity that gets progressively lower at higher iron content (A99, A114, A114-D122Y, Figure 4A,C,E).

The signal loss is less marked for the D122Y pathological variant than for the WT: at a 1:50 FXN: Fe^2+^ ratio, the signal loss for A114C-D122Y (and for A99C) is 40%, while that for A114C raises to 65%. For the A114 position the signal loss is accompanied by a change in shape, whereas no change is observed for the A99 position: this difference arises from the intrinsic mobility of the probe sidechain in the two positions and has been discussed more in-depth in the Supporting Information (Figures S5 and S6). The signal loss is independent of the presence of Ficoll (data not shown), and we verified that it is caused by a redox reaction of the M-TETPO nitroxide with Fe^2+^ as observed for MTSSL. To test this hypothesis, we removed protein-bound Fe^2+^ by treatment with 1,10-phenanthroline (PHEN) which forms a strongly colored red complex with Fe^2+^. Treating the sample with a three-fold molar excess of PHEN relative to iron leads to quantitative removal of Fe^2+^ from FXN, as evaluated spectrophotometrically by UV-Vis under anaerobic conditions from the absorption band at λ_max_ = 508 nm (see Supporting Information, Figure S7) [40]. When stripping Fe^2+^ with PHEN from FXN, the solution turns red but the EPR signal is not restored, confirming that an irreversible reduction of the nitroxide has taken place.

We can then hypothesize that the signal loss is directly correlated with the presence of a nearby Fe^2+^ binding site. Indeed, signal loss does not originate from Fe^2+^ free in solution, as confirmed by the lack of effect in the three mutants labeled at positions far away from the iron-binding region. When a ferrous iron is close to the nitroxide of the spin label, the redox reduction can be favored by the vicinity between the redox partners, and nearby amino-acid residues might also favor the electron transfer kinetics. The exact mechanism will be tested in a future work; nevertheless, the redox reaction is a clear sign of proximity and this makes M-TETPO an interesting probe able to detect nearby Fe^2+^ binding sites.

### 2.4. Fluorescence Spectra of FXN Interacting with Fe^3+^

**Figure 5:**
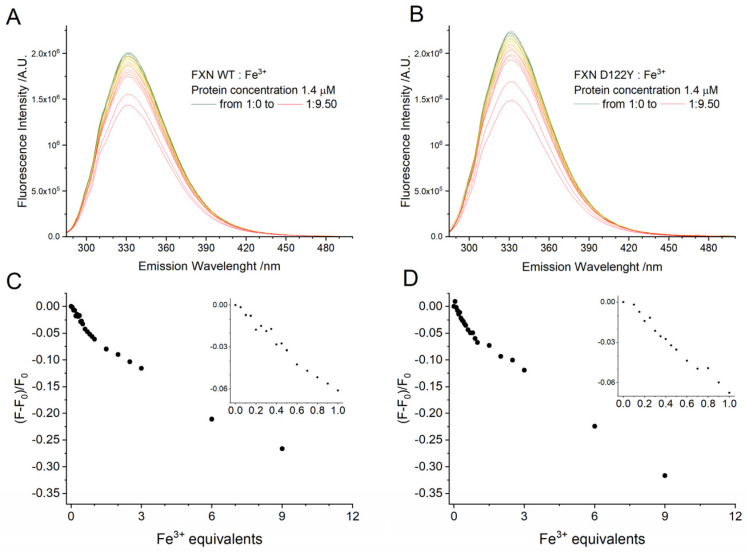
Tryptophan fluorescence spectra of WT FXN and D122Y variant at increasing amounts of Fe^3+^. Protein concentration 1.4 µM, 288 K, λ_exc_ = 280 nm, λ_max_ = 332 nm. (**A**,**B**) Fluorescence of FXN WT and D122Y, respectively; (**C**,**D**) fluorescence quenching by Fe^3+^ calculated at λ_max_ for FXN WT and D122Y, respectively; in the inset, a zoom of the sub-stoichiometric region.

The quenching effect of Fe^3+^ addition to the fluorescence emission of the three Trp residues of wild type and D122Y FXN proteins was explored. Relative to what was previously reported [29], here we worked at a seven-fold lower protein concentration ([FXN] = 1.4 µM) and with a greater focus on the sub-stoichiometric region since, as shown by the EPR experiments reported above, aggregation starts to occur when adding more than few equivalents. The fluorescence signals and their quenching are shown in Figure 5; quenching is reported as (F − F_0_)/F_0_, where F is the fluorescence intensity at the maximum of emission at a given amount of quencher and F_0_ the intensity in the absence of quenchers. The normalized signal shapes of the WT and the D122Y FXN are identical, as expected (reported in the Supporting Information, Figure S8). In addition, the normalized emission profiles in the absence of iron and at 9.5 equivalents of iron do not show any difference neither in the position of the maxima nor in the shape of the band, indicating that the three Trp residues are equally quenched by the presence of Fe^3+^. The quenching of Trp fluorescence by Fe^2+/3+^ is likely due to an energy transfer mechanism, which is a distance dependent process [41]. Due to the short distance between Trp residues and to the homo-FRET, the observed quenching of the fluorescence could be due only to Fe^2+/3+^ ions close to any of the Trp residues.

The signal decreases sharply in the sub-stoichiometric region, levels off somewhat around 1.5 equivalents of Fe^3+^ and then falls off more sharply again at six equivalents and beyond. Visual inspection of the cuvette revealed no visible precipitate in the sub-stoichiometric region, but substantial precipitate already at six equivalents. It is, therefore, impossible to reliably assess a binding constant for Fe^3+^ with human FXN using fluorescence due to the presence of Fe^3+^-induced FXN aggregates (in agreement with the EPR results reported above). The WT and the D122Y variant show almost no difference in the quenching profile.

### 2.5. Fluorescence Spectra of FXN Interacting with Fe^2+^

The quenching effect of Fe^2+^ addition to the fluorescence emission of WT and D122Y FXN was explored using strictly anaerobic solutions at low concentration ([FXN] = 1.4 µM) and focusing on the sub-stoichiometric region. To ensure that the Fe^2+^ is not oxidized during the experiment, both the iron stock solution and the experimental cuvette were filled under nitrogen atmosphere and closed with silicon septa. The experimental compartment was flushed with nitrogen at all times. Fe^2+^ was injected into the cuvette through the septum using a Hamilton syringe flushed with nitrogen before any addition. The absorption of a stock solution of Fe^2+^ was checked and found it does not contribute significantly in the experimental conditions (λ_exc_ = 280 nm).

**Figure 6:**
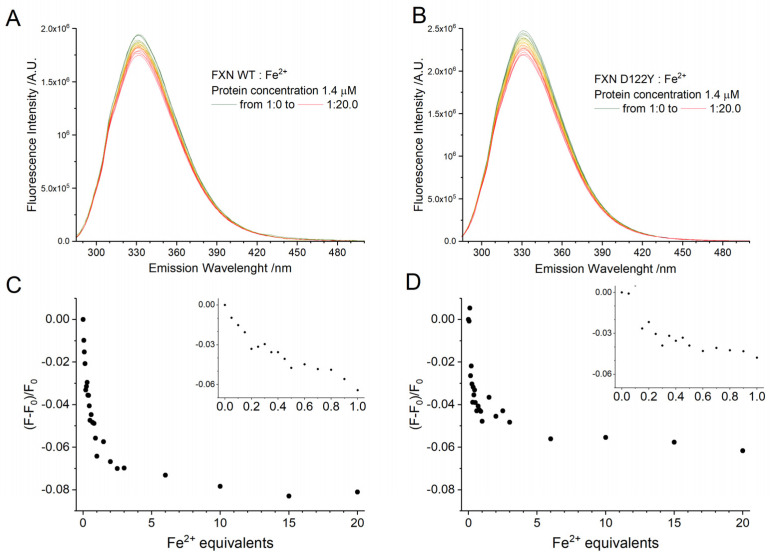
Tryptophan fluorescence spectra of WT FXN and D122Y variant at increasing amounts of Fe^2+^. Protein concentration 1.4 µM, 288 K, λ_exc_ = 280 nm, λ_max_ = 332 nm. (**A**,**B**) Fluorescence of FXN WT and D122Y, respectively; (**C**,**D**) fluorescence quenching by Fe^2+^ calculated at λ_max_ for FXN WT and D122Y, respectively; in the inset, a zoom of the sub-stoichiometric region.

The fluorescence signals and their quenching, expressed as (F − F_0_)/F_0_, are reported in Figure 6. As discussed above, the quenching from Fe^2+^ has the same mechanism as the one from Fe^3+^, i.e., through an energy transfer mechanism from a bound iron ion to the nearest of the three Trp residues. From the normalized spectra (reported in the Supporting Information, Figure S8), Fe^2+^ does not alter the shape of the emission profiles even at 20 equivalents, indicating that all three Trp residues are equally quenched as for the case of Fe^3+^.

The signal decreases sharply in the sub-stoichiometric region, levels off somewhat around 2.2 equivalents of Fe^2+^, and then does not further decrease reaching a plateau. The absence of further quenching indicates that either Fe^2+^ populates only one binding site, or that the other binding sites are too far from the Trp to further quench them as further discussed below. This is true only in rigorously anaerobic conditions: in a control experiment in the presence of oxygen, quick conversion of Fe^2+^ to Fe^3+^ leads to a progressively increasing quenching (data not shown).

The WT shows a markedly more intense quenching than the D122Y variant, the (F − F_0_)/F_0_ being −0.08 vs. −0.06 at the 20 equivalents. The absolute quenching for Fe^2+^ in the sub-stoichiometric region is comparable for ferrous and ferric iron (about −0.08 for both oxidation states). Proceeding with the titration, at 10 equivalents the (F − F_0_)/F_0_ for WT is −0.08 for Fe^2+^ much lower than the quenching observed for Fe^3+^, −0.27. The titration curves have been analyzed according to a literature method [42], the details of the analysis are reported in the Supporting Information (see Figure S9); we obtained K_D_ = 4.2 × 10^−7^ for the WT and K_D_ = 7.8 × 10^−7^ for the D122Y variant, and for both cases a stoichiometry of one strongly bound Fe^2+^. These values must be considered only indicative, due to the low concentration used for the experiments which introduces a 10% error in the estimate. 

### 2.6. CD Spectra of FXN Interacting with Fe^3+^ and Fe^2+^

**Figure 7:**
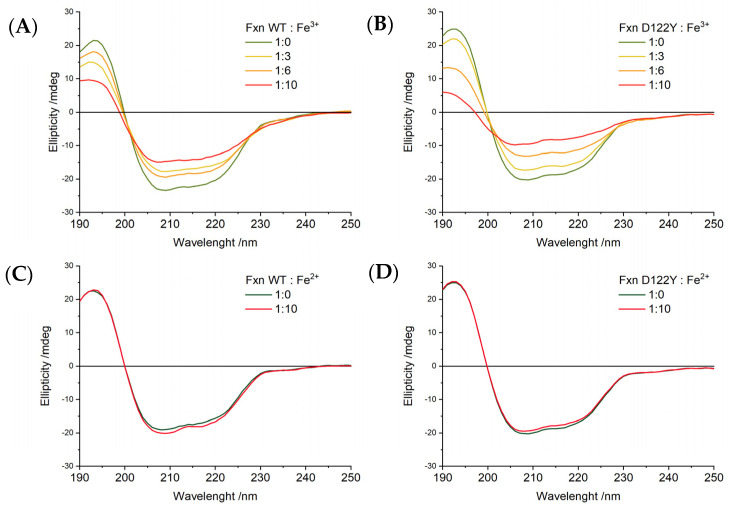
CD spectra of FXN of WT FXN and D122Y variant with either Fe^3+^ or Fe^2+^. Protein concentration 50 µM. (**A**) WT FXN with increasing amounts of Fe^3+^; (**B**) D122Y FXN with increasing amounts of Fe^3+^; (**C**) WT FXN with Fe^2+^; (**D**) D122Y FXN with Fe^2+^.

The effects of iron binding on the secondary structure of human wild type and D122Y FXN proteins were evaluated by circular dichroism spectroscopy, as reported in Figure 7. In the absence of iron, the CD spectra for both WT and D122Y FXN are identical and show the typical shape of a mixed alfa-beta secondary structure [43].

Increasing addition of Fe^3+^ leads to a progressive loss of ellipticity across the whole spectral range for both proteins, Figure 7A,B. There are no marked changes in spectral shape and no well-defined isodichroic point can be observed; in addition, at high Fe^3+^ amounts, the low wavelength region shows signs of increased scattering. It was previously shown that a peptide derived from the main helix of FXN can bind one Fe^3+^ ion through its acidic residues and gains alpha-helical structure upon binding [44]. All these observations suggest that the loss of ellipticity cannot be interpreted as a loss of structure, but only as a sign of protein aggregation/precipitation as already proven by EPR spectroscopy. 

On the contrary, additions of up to ten equivalents of Fe^2+^ do not change the CD spectra of WT and D122Y FXN, Figure 7C,D. Therefore, Fe^2+^ binding to FXN does not induce either significant secondary structure changes or protein aggregation.

## 3. Discussion

FXN has been proposed to have several physiological roles: allosteric regulator of ISC biogenesis [21,45], the iron source for building ISC clusters [24], regulator of iron homeostasis [25] and ferroptosis [26]. All the above roles involve iron ions; therefore, it is of key importance to understand the effects of ferrous and ferric iron on the structure and function of FXN. In this work, the response of human FXN, both WT and D122Y variant (the only pathological variant with a mutation in the iron-binding region), was investigated in the presence of ferrous and ferric ions using the SDSL EPR technique, which has never been used on FXN before, and the results were combined with those obtained by fluorescence and circular dichroism. 

Ferrous iron binds human FXN without causing any large secondary structure changes as shown by CD, even at a 10-fold excess. On the other hand, EPR clearly indicates that Fe^2+^ binding does not alter local backbone dynamics of regions that are not involved in iron binding, as revealed by the lack of mobility changes of the spin probes at all the sites investigated. Moreover, no FXN oligomerization occurs in the presence of Fe^2+^, which is in agreement with previous results [30]. The M-TETPO probe that we adopted, was liable to reduction by Fe^2+^ bound in close proximity (at sites A99 and A114), thus proving that Fe^2+^ binds on both ends of the long alpha helix, in accordance with previous reports [15,29,35]. The majority of the EPR probe reduction takes place in the first three iron equivalents. The EPR results also put the results of fluorescence quenching into context. Fluorescence analysis leads to the conclusion that at least one strongly bound iron ion can be located in the long helix containing many Asp and Glu residues, close to W168/W173 (note that W155 is located in a region where putative Fe^2+^ binding sites are absent). This would be the site that reduces the spin label at position 99 (which is close to these two Trp). Conversely, the fluorescence measurements are not sensitive to binding of the Fe^2+^ ions in regions which are far from the Trp. Indeed, the Fe^2+^ binding site at the end of the helix suggested by the EPR spectra of A114C may escape fluorescence detection being out of range of the energy transfer Trp quenching mechanism. In conclusion, the number and the position of Fe^2+^ binding sites cannot be easily determined based on fluorescence quenching only, due to the presence of multiple Trp emitters spatially close to each other, and our results explain the apparent conflict in the number of iron binding sites reported in the literature. 

In contrast to ferrous iron, ferric iron binding promotes FXN aggregation when few equivalents are added as shown EPR and CD experiments while the initial binding events at sub-stoichiometric ratios do not induce it. The WT protein exhibited a loss of ellipticity upon Fe^3+^ addition, and centrifugation of EPR samples showed the precipitation of the immobilized portion of the sample. We observed immobilization at sites placed not only in the long acidic alfa helix, which is the major putative iron binding region, but also in the second beta strand and the shorter helix. Thus, the interactions among monomers involve all the secondary structure elements and a large surface area is interested in the aggregation induced by iron binding: as such, it is likely that FXN does not form dimers, rather oligomers, and these are arranged in a globular fashion, without a preferential interface. Immobilization is the result of the constraint of the local motion of the labels, arising from new contacts between different protein monomers upon aggregation. Fe^3+^-mediated oligomerization was previously suggested using SAXS, light scattering, and electron microscopy, at least at high FXN concentration (150–700 µM), with EM images suggesting that at least a portion of the aggregates are present as ring tetramers [33]. This was in contrast to previous reports, according to which human frataxin showed no tendency towards oligomerization, while the correspondent yeast and *E. coli* orthologs aggregate in the presence of both Fe^2+^ and Fe^3+^ [34]. Since Fe^3+^ promotes the formation of FXN aggregates, also at relatively low protein concentration, it is impossible to evaluate the dissociation constant of Fe^3+^ using fluorescence or calorimetry since the quenching or heat, respectively, have dominant contributions from aggregation and precipitation phenomena. Based on our results, the data previously reported for the dissociation constants and binding stoichiometry of human FXN with Fe^3+^ need to be critically reconsidered [22]. 

Our results may have some implication in the context of the physiological behavior of FXN. The lack of perturbation of the structure of FXN by Fe^2+^ suggests that ferrous iron might easily bind/unbind FXN without any change in structure and dynamics. Therefore, FXN might partake in the ISC machinery while exchanging Fe^2+^ from the solution to the complex. With respect to the aggregation induced by Fe^3+^, we showed that it is strongly dependent on FXN concentration, thus large oligomers are not expected to be present at the low physiological concentrations of FXN in normal physiological states (FXN mitochondrial concentration is estimated to be between 0.1–1.0 μM in *S. cerevisiae*, by combining literature data [46,47]). However, in FRDA patients, a significant iron imbalance was observed [48,49,50], and pathological conditions are likely to trigger oligomerization. It seems unlikely, then, that the interaction of FXN with Fe^3+^ has no physiological function and reflects only a non-specific electrostatic effect. Indeed, a recent report linked FXN deficiency to cell death by ferroptosis [26]. Therefore, in conditions of an excess of ferric iron, a contribution of FXN to iron scavenging could be important, substantiating the function of FXN as a ‘ferritin-like’ scavenger helping iron homeostasis by keeping iron in a nontoxic, soluble and more bio-available form [51]. In this context, it is important to note that, as demonstrated by the effects of EDTA, the aggregation is reversible: therefore, once the imbalances in cell physiology that are responsible for the increase in ferric iron are overcome, FXN can potentially return monomeric. 

Finally, the use of SDSL EPR allowed us to highlight differences in iron binding between the WT and the D122Y variant. A lower iron affinity of the D122Y FXN has been related before to a higher intrinsic mobility of the iron-binding region of the protein [52]. Indeed, an increased mobility of D122Y FXN in comparison with WT FXN is proved by the M-TETPO spin probe at the A114 position. The D122Y variant shows lower EPR signal loss in the presence of Fe^2+^ relative to the WT, and reduced fluorescence quenching, confirming a lower Fe^2+^ affinity. In addition, since the M-TETPO probe is sensitive to bound Fe^2+^ in the vicinity, the lower signal loss observed in the D122Y variant implies that the aspartate side chain in position 122 is directly involved in ferrous iron binding. In contrast, in the sub-stoichiometric region, the effects of Fe^3+^ on the fluorescence quenching show no difference between the WT and the D122Y variant. The differences in fluorescence quenching by Fe^3+^ between the WT and D122Y that were previously observed [29,52] are referred especially to the region dominated by precipitation, and as such depend strongly on the protein concentration as already described. Overall, the different binding affinity with ferrous, but not ferric, iron likely arises from a combination of variations in the structural dynamics of the region and loss of the aspartate partaking in iron binding. Since it was previously observed that D122Y variant retains some capability to enhance the desulfurase activity *in vitro* [29], it is likely that the pathological nature of D122Y lies in a combination of lowered ability of the variant to participate in Fe^2+^ trafficking and less than optimal conformation hampering the interaction with the other proteins in the ISC cluster biogenesis complex.

## 4. Materials and Methods

### 4.1. Materials

The spin label MTSSL, (1-Oxyl-2,2,5,5-tetramethyl-∆3-pyrroline-3-methyl) Methanethiosulfonate, was purchased from Toronto Research Chemicals. M-TETPO was synthesized as described below. Unless otherwise specified, the buffer composition for spectroscopic experiments was: 25 mM HEPES (pH 7.0) 50 mM KCl. Ferrous iron solutions were prepared in anaerobic atmosphere in degassed deionized water slightly acidified starting from (NH_4_)_2_Fe(SO_4_)_2_·6H_2_O (Mohr’s Salt, >99% Fe^2+^ content). Ferric iron solutions were prepared from FeCl_3_·6H_2_O in HCl at pH = 0.8. Ficoll^®^ PM 70 stock solution was prepared dissolving the polymer in degassed buffer at a 35% *w/w*% concentration, under anaerobic atmosphere. 1,10-Phenanthroline monohydrate was dissolved in dilute buffer. Unless otherwise specified, chemicals were purchased from Merck and used without further purification.

### 4.2. Synthesis of M-TETPO

**Scheme 2:**
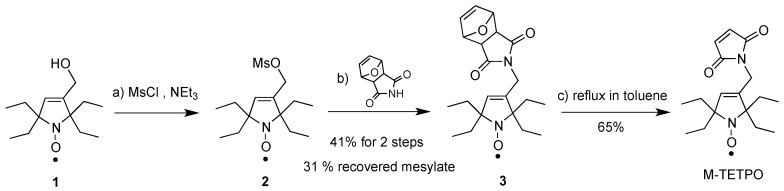
Synthetic way to prepare TETPO from alcohol 1. (**a**) MsCl, Et_3_N, DCM 0 °C to RT, 2 h; (**b**) protected maleimide, K_2_CO_3_, DMF; (**c**) reflux, toluene.

The synthesis of M-TETPO is reported in Scheme 2; further details are reported in the Supporting Information (pages S12–13). It was prepared in three steps from alcohol **1**, which was synthesized according to a published procedure [53]. Thus, reaction of alcohol **1** with mesyl chloride (MsCl) and triethylamine (NEt_3_), followed by the substitution of the crude resulting mesylate **2** with protected maleimide, afford compound **3** in 41% yield and the unreacted mesylate **2** in 31% yield. Finally, retro-Diels-Alder was performed with compound **3** by refluxing in toluene to afford pure M-TETPO in 65% yield. Analytical data were identical to those reported for TETPO [39].

### 4.3. Heterologous Expression and Purification of Human Wild Type and D122Y FXN Proteins 

A plasmid containing the coding sequence of human wild type mature FXN, i.e., pET-9b/FXN (90–210), was previously obtained in our laboratory [54]. FXN mutants were obtained through site-directed mutagenesis with the QuickChange^®^ II Site-Directed Mutagenesis Kit (from Agilent Technologies), using as template the pET-9b/FXN (90–210) plasmid and the couples of primers listed in the Supporting Information (see the Table S1 at page S2). Each sequence was checked by DNA sequencing (at GATC Biotech, Germany). *Escherichia coli* BL21 (DE3) cells were chemically transformed with the plasmids of interest and positive clones were selected by antibiotic resistance. The expression of the wild type and mutant proteins was induced by adding 1 mM isopropyl-β-thiogalactopyranoside (IPTG) in LB medium and incubating the bacteria cultures at 30 °C overnight at constant stirring. Cells were then harvested by centrifugation at 5000× *g* and 4 °C for 20 min, resuspended in lysis buffer 25 mM HEPES (pH = 7.0) supplemented with protease inhibitors (1 μg/mL pepstatin A, 1 μg/mL leupeptin, 1 μg/mL antipain, 100 μM PMSF) and lysed by French press. The supernatant fractions were isolated from cell debris by centrifugation at 17,000× *g* and 4 °C for 15 min and incubated with EDTA 10 mM for 1 h at 4 °C, in gentle agitation. Proteins were purified combining anionic exchange and size exclusion chromatographies. The first chromatographic step was performed using a cationic DEAE (Diethylaminoethyl) Sepharose column using 25 mM HEPES (pH = 7.0) as buffer of equilibration and 25 mM HEPES (pH = 7.0), 1 M KCl as elution gradient buffer. The fractions corresponding to frataxin, as assessed by SDS PAGE, were collected, pooled together, concentrated by centrifugal filters (Amicon Ultra Centrifugal Filter, 3000 NMWL, from Merck Millipore, Burlington, MA, USA) and purified by size-exclusion chromatography (SEC). For FXN mutants containing cysteines, an incubation with 1 mM of dithiothreitol (DTT) for 1 h at 4 °C has been performed after cationic exchange. The second purification step was performed on a Superdex 200 GL 10 300 column (from GE Healthcare), equilibrated in a buffer containing 25 mM HEPES (pH = 7.0) and 50 mM KCl. To estimate the molecular weight of the protein samples, the column was equilibrated in the same buffer and calibrated with the standards thyroglobulin (669 kDa), ferritin (440 kDa), β-amylase (200 kDa), bovine serum albumin (67 kDa), carbonic anhydrase (29 kDa) and cytochrome *c* (12 kDa). The eluted fractions containing frataxin proteins were finally pooled together and the molar concentration of the protein samples was determined spectroscopically using ε_280nm_ = 26930 M^−1^cm^−1^ for all mutants and ε_280nm_ = 28420 M^−1^cm^−1^ for those containing D122Y point mutation (molar extinction coefficients evaluated by ExPASy ProtParam tool). Protein purity and integrity were finally assessed by 15% SDS-PAGE and Coomassie blue staining, prior to any spectroscopic experiment reported in this work.

### 4.4. Spin Labeling

Protein samples were labeled with MTSSL or M-TETPO for EPR experiments. Samples labeled with MTSSL were obtained by adding to the purified protein (at a concentration of about 150 μM) a sevenfold molar excess of spin label (dissolved in DMSO) and incubating the protein at 4 °C overnight in the dark and gentle stirring. Excess of non-ligated spin label was removed from the protein by PD10 desalting column (GE Healthcare) using 25 mM HEPES (pH = 7.0) and 50 mM KCl as final buffer. For the experiments with Fe^2+^, in order to avoid possible oxidation of iron, anaerobic conditions have been adopted in the labeling with MTSSL and all steps were performed under a glove box. For the labeling with M-TETPO, purified proteins (at a concentration of 50 μM) were previously incubated with DTT in a molar ratio of 1:100 at 4 °C for 30 min in slow agitation. The excess of DTT was removed from the samples by PD10 desalting column (GE Healthcare) using 25 mM HEPES (pH = 7.0) and 50 mM KCl as elution buffer. Proteins were labeled with a tenfold molar excess of M-TETPO (dissolved in acetonitrile) and incubated at 4 °C for 2 h in the dark and slow agitation. Labeled protein samples were concentrated by centrifugal filters (Amicon Ultra Centrifugal Filter, 3000 NMWL, from Merck Millipore, Burlington, MA, USA) and the excess of spin label was then removed by gel filtration using a Superdex 200 GL 10 300 column (from GE Healthcare) and 25 mM HEPES (pH = 7.0), 50 mM KCl as elution buffer. MTSSL/M-TETPO-labeled proteins were finally concentrated by centrifugal filters to a volume suitable for EPR spectroscopic analysis, and their concentration determined by UV-Vis spectroscopy as previously described.

### 4.5. Electron Paramagnetic Resonance (EPR) Spectroscopy

EPR spectra were recorded on an ELEXSYS E580 spectrometer equipped with a SHQ cavity, both from Bruker, Germany. The experiments were performed at room temperature, typically using the following parameters: microwave frequency 9.87 GHz, microwave power 10 mW (attenuation 12 dB), sweep width 150 mT, center field 352 mT, conversion time 164 ms, time constant 82 ms, modulation amplitude 1.6 mT, 1024 points, 3–25 averages (depending on protein concentration). Samples were prepared thoroughly mixing 32 µL of Ficoll PM70 stock solution (or buffer when needed), 7 µL of protein stock solution, and 1 µL of Fe^2+^ or Fe^3+^ solution; the resulting solution was put in glass capillary (internal diameter 0.8 mm) and measured under nitrogen gas flow. The final protein concentration was typically 50 or 10 µM for Fe^2+^ or Fe^3+^ respectively, save for when differently stated. The different FXN:Fe^2+^ or Fe^3+^ molar ratios were prepared from an appropriate dilution of a concentrated stock solution.

### 4.6. Simulation of EPR Spectra

The simulation of the EPR spectra allows getting quantitative information on the mobility of the spin label and eventually pointing out the presence of multiple components. To perform the simulations, one must know or estimate the nitroxide g-tensor (**g**) and hyperfine tensor (**A**) and then adopt a model of the spin label motion based on the stochastic Liouville equation, Microscopic Order Macroscopic Disorder (MOMD) being the one used most often [55]. MOMD was developed considering that the spin label molecular motion is limited by the protein structure (microscopic order) and that the macromolecules adopt all the possible orientations with respect to the external magnetic field (macroscopic disorder). The MOMD model makes it possible to describe the spin label mobility in terms of the diffusion tensor **D**, its orientation relative to the nitroxide frame expressed by the Euler angles Ω_D_ (but usually reduced to a single angle β_D_ for symmetry), and the order parameter S. The mobility is often discussed in terms of the rotational correlation time τ_c_, which is derived from the diffusion tensor: for a nitroxide characterized by an axial diffusion τ_c_ *=* 1/6(*D_||_D_⊥_*)^1/2^. We used the MultiComponentEPR827.vi program by Christian Altenbach to perform the simulations. The program is written in LabVIEW (National Instruments) and can be freely downloaded from the following website: http://www.biochemistry.ucla.edu/Faculty/Hubbell/. 

### 4.7. Fluorescence

Fluorescence experiments were performed on a FLS 1000 UV/Vis/NIR photoluminescence spectrometer by Edinburgh Instruments with a 450 W Xenon Arc lamp for excitation at 285 nm and a PMT-980 detector. The Peltier controlled holder allowed measuring at 288 K under stirring. The sample compartment was under constant nitrogen flow to avoid condensation on the windows and keeping an anaerobic atmosphere. Experiments were conducted using a fluorescence cuvette (117104F-10-40 from Hellma, Mülheim, Germany) with 10 × 4 mm optical path length and gas tight screw cap with a silicon septum for addition of the ferrous/ferric iron solutions via a gas tight microsyringe (from Hamilton Company, Reno, NV, USA) under anaerobic atmosphere. Proteins were used at 1.4 µM concentration.

### 4.8. Circular Dichroism

CD measurements were performed with a Jasco J-1500 spectropolarimeter. Experiments were performed at 298 K using a Jasco PTC-423 Peltier cell holder connected to a Jasco PTC-423S Peltier controller. Far-UV CD spectra were collected using a cylindrical cell (121-0.20-40 from Hellma) with 0.2 mm optical path length using 50 µL of protein solution. Data were acquired at a scan speed of 20 nm/min and at least three scans were averaged. Proteins were used at a concentration of 50 µM, in a 0.5 mM Tris-HCl pH 7.0, 1 mM KCl buffer. For experiments with ferrous iron, the cuvette was filled and sealed under anaerobic atmosphere in a glove box; the compartment of the CD spectropolarimeter is anaerobic since it is constantly under nitrogen flow.

## 5. Conclusions

The results obtained through the combination of SDSL EPR, fluorescence and CD spectroscopies prove that human FXN interacts with Fe^2+^ and Fe^3+^ with a markedly different outcome. Fe^3+^ causes aggregation in a concentration-dependent fashion, starting when adding few equivalents, making difficult to evaluate the exact binding stoichiometry. Fe^2+^, the physiological form of iron in cells, simply binds without affecting FXN conformation or inducing aggregation. 

We suggest that both ions might be relevant in the context of the proposed FXN physiological functions. FXN role as a regulator of iron homeostasis and ferroptosis might be exerted by sequestering the excess of ferric iron through concentration dependent reversible aggregation. The absence of structural and dynamic effects of Fe^2+^ binding suggests that FXN is able to partake in ferrous iron trafficking even while bound to the ISC cluster assembly machinery, possibly intercepting the iron from the labile iron pool in solution and passing it to ISCU during cluster formation. The different binding affinity with ferrous, but not ferric, iron in D122Y pathological shows that the aspartate side chain in position 122 is directly involved in ferrous iron binding and that the mobility of the protein in the vicinity of the mutated site is increased compared to the WT. Both the effects may be related to the pathological nature of D122Y.

The next step will involve exploring the effects of Fe^2+^/Fe^3+^ on FXN structure and dynamics in the presence of the other partners of the ISC cluster assembly machinery, ISCU, NFS1 and the accessory proteins. We will use the currently available spin labeled sites plus new others strategically chosen in light of the available structure of the complex.

## Figures and Tables

**Table 1:** The rotational correlation times of the different FXN labeled positions in 28% *w*/*w* Ficoll solution. The g tensor principal components are: g_xx_ = 2.0088, g_yy_ = 2.0070, g_zz_= 2.0030. The hyperfine tensor principal components are: A_xx_ = 0.79 mT; A_yy_ = 0.54 mT; A_zz_ = 3.68 mT. The spectrum of A193C at a FXN:Fe^3+^ 1:50 ratio is characterized by two components.

	FXN:Fe^3+^ 1:0			FXN:Fe^3+^ 1:50		
Position	β_D_	S	τ_c_	β_D_	S	τ_c_
A114C	17°	0.44	0.7 ns	0°	0.53	5.3 ns
A114C/D122Y	17°	0.44	0.7 ns	0°	0.53	5.3 ns
T133C	20°	0.36	0.9 ns	0°	0.53	3.8 ns
H183C	42°	0.44	3.9 ns	0°	0.53	8.4 ns
A193C	45°	0.44	1.8 ns	0°	0.53	3.6 ns
				0°	0	2.2 ns

## Supplementary Materials

Supplementary materials can be found online at https://www.mdpi.com/1422-0067/21/24/9619/s1.
