# Supplementary material for: Effects of Fe2+/Fe3+ Binding to Human Frataxin and Its D122Y Variant, as Revealed by Site-Directed Spin Labeling (SDSL) EPR Complemented by Fluorescence and Circular Dichroism Spectroscopies
Source: Int J Mol Sci. 2020 Dec 17;21(24):9619. doi: 10.3390/ijms21249619 (PMC7766144; doi:10.3390/ijms21249619)
Supplement: Supplementary file 1 [file ijms-21-09619-s001.pdf]

Supplementary Information

for

Effects of Fe^{2+}/Fe^{3+} binding to human frataxin and its D122Y variant, as revealed by Site Directed Spin labelling (SDSL) EPR complemented by Fluorescence and Circular Dichroism spectroscopies

Davide Doni[1], Leonardo Passerini[2], Gérard Audran[3], Sylvain R. A. Marque[3], Marvin Schulz[3], Javier Santos[4,5], Paola Costantini[1], Marco Bortolus[2]* and Donatella Carbonera[2]

[1] Department of Biology, University of Padova, Viale G. Colombo 3, 35131 Padova, Italy

[2] Department of Chemical Sciences, University of Padova, Via F. Marzolo 1, 35131 Padova, Italy

[3] Aix Marseille Universitè, CNRS, ICR, UMR 7273, case 551, Ave Escadrille Normandie Niemen, 13397 Marseille Cedex 20, France

[4] Instituto de Biociencias, Biotecnología y Biomedicina (iB3-UBA). Departamento de Química Biológica, Facultad de Ciencia Exactas y Naturales, Universidad de Buenos Aires. Intendente Güiraldes 2160 - Ciudad Universitaria, 1428EGA CONICET, Godoy Cruz 2290, C1425FQB. C.A.B.A. Argentina·

[5] Instituto de Química y Fisicoquímica Biológicas, Dr. Alejandro Paladini, Universidad de Buenos Aires, CONICET, Junín 956, 1113AAD C.A.B.A., Argentina

*to whom correspondence should be addressed: marco.bortolus@unipd.it

Table of contents:

List of primer	S2
Analysis of immobilized FXN	S3
Centrifugation experiment	S4
Reversibility of Fe^{3+} aggregation via chelation by EDTA	S5
M-TETPO labelled FXN with Fe^{3+}	S6
Sidechain conformer analysis of A99C and A114C labelled with M-TETPO	S7-8
Removal of bound Fe^{2+} via chelation by PHEN	S9
Normalized fluorescence spectra	S10
Fe^{2+} fluorescence quenching analysis	S11
Synthesis of M-TETPO	S12-13

Table S1 List of primer

FXN mutant	Primer name	Primer sequence
FXN_D122Y	FXN_D122Y_for	5’ - GCCATACACGTTTGAGTACTATGATGTCTCCTTTGG - 3’
	FXN_D122Y_rev	5’ – CCAAAGGAGACATCATAGTACTCAAACGTGTATGGC – 3’
FXN_A99C	FXN_A99C_for	5’ - GAGACCACCTATGAAAGACTATGCGAGGAAACGCTGGACTC – 3’
	FXN_A99C_rev	5’ – GAGTCCAGCGTTTCCTCGCATAGTCTTTCATAGGTGGTCTC – 3’
FXN_A114C	FXN_A114C_for	5’ - CAGAGTTTTTTGAAGACCTTTGCGACAAGCCATACACGTTTGAG – 3’
	FXN_A114C_rev	5’ – CTCAAACGTGTATGGCTTGTCGCAAAGGTCTTCAAAAAACTCTG – 3’
FXN_T133C	FXN_T133C_for	5’ - TCCTTTGGGAGTGGTGTCTTATGTGTCAAACTGGGTGG – 3’
	FXN_T133C_rev	5’ – CCACCCAGTTTGACACATAAGACACCACTCCCAAAGGA – 3’
FXN_H183C	FXN_H183C_for	5’ - CGGCGTGTCCCTCTGTGAGCTGCTGGCC – 3’
	FXN_H183C_rev	5’ – GGCCAGCAGCTCACAGAGGGACACGCCG – 3’
FXN_A193C	FXN_A193C_for	5’ - GGCCGCAGAGCTCACTAAATGCTTAAAAACCAAACTGGAC – 3’
	FXN_A193C_rev	5’ – GTCCAGTTTGGTTTTTAAGCATTTAGTGAGCTCTGCGGCC – 3’

Analysis of immobilized FXN

The gradual spectral changes upon Fe^{3+} addition have been analyzed as combination of the two "pure" contributions shown in Figure S1 left:

1) the mobile nitroxide lineshape obtained from the A193C in the absence of Fe^{3+}, red spectrum;

2) the immobilized lineshape obtained from the A193C at 100 μM with a FXN:Fe^{3+} 1:50 ratio, pink spectrum.

A weighted sum of the two components (previously normalized to the same number of spins by dividing each spectrum by its double integral) has been performed to reproduce the spectra at various FXN concentrations and FXN:Fe^{3+} molar ratios. A full example is reported in the right panel of Figure S1.

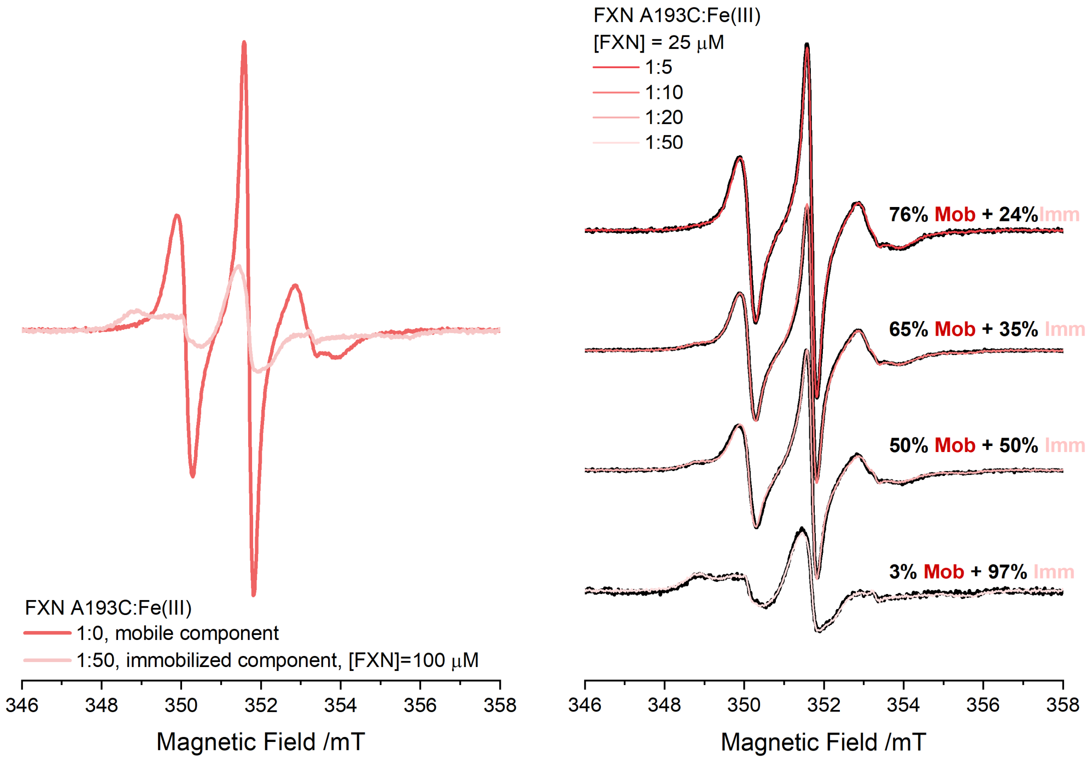

Figure S1 Left, the experimental spectra of A193C in the absence, mobile component (red) and in the presence of Fe(III) at a FXN:Fe^{3+} 1:50 ratio, immobilized component (pink); [FXN] = 100 μM. Right, experimental spectra (black) and simulations (color) obtained as a weighted sum of the components on the left.

Centrifugation experiment

A193C mutant labelled with MTSSL in buffered solution (no Ficoll) was incubated at a FXN:Fe^{3+} 1:20 ratio, [FXN] = 10 μM. The lineshape, shown in Figure S2, shows a partial averaging of the different components relative to the sample in Ficoll, as expected from the fast tumbling of FXN. Nevertheless, the immobilized component is visible as indicated by the arrows (black spectrum). Upon centrifugation of the sample, the EPR spectrum of the supernatant shows only the mobile component, indicating that the immobilized component has been precipitated.

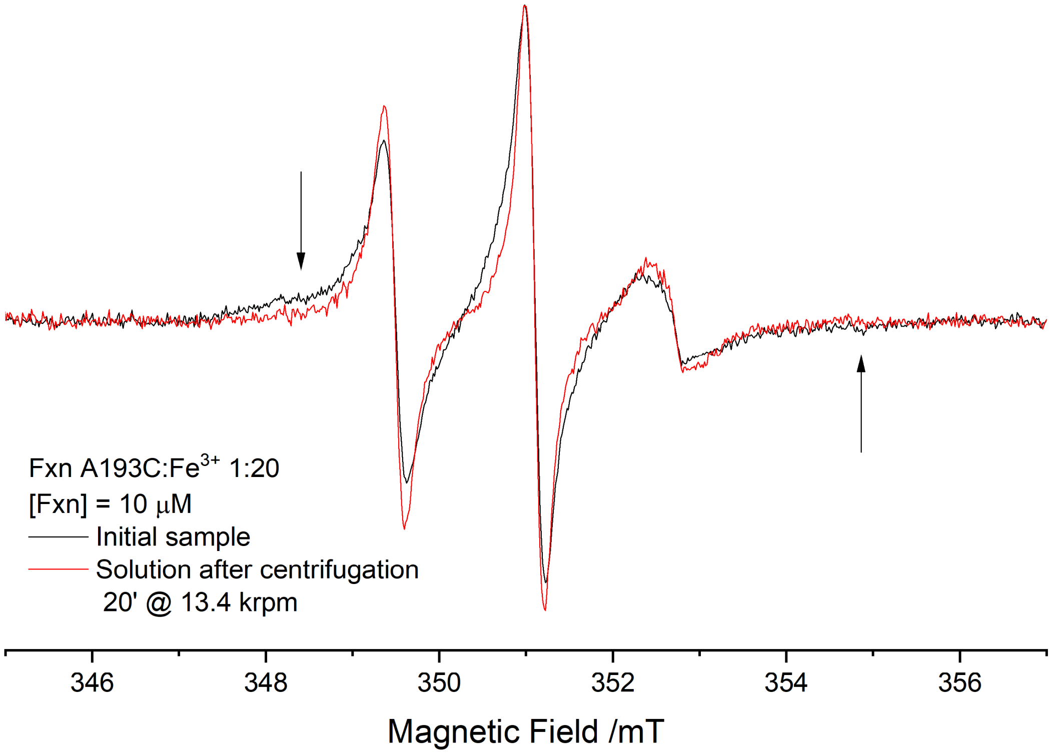

Figure S2 The experimental spectra of A193C in buffer solution at a FXN:Fe^{3+} 1:20 ratio, [FXN] = 10 μM. The figure shows the spectrum before (black) and after (red) centrifugation. The arrows indicate the features of the immobilized component.

Reversibility of Fe^{3+} aggregation via chelation by EDTA

The reversibility of FXN aggregation was checked by incubating the sample with A193C at a 1:20 FXN:Fe^{3+} molar ratio, taking the EPR spectrum before (red) and after (pink) incubation of the sample with a three-fold molar excess of EDTA relative to iron, as can be seen from Figure S3 left. The lineshape partially reverts to the one in the absence of Fe^{3+}, showing that aggregation is at least partially reversible. As a control that EDTA does not perturb the protein, we report the spectra of the protein with/without EDTA in the absence of Fe^{3+}, Figure S3 right.

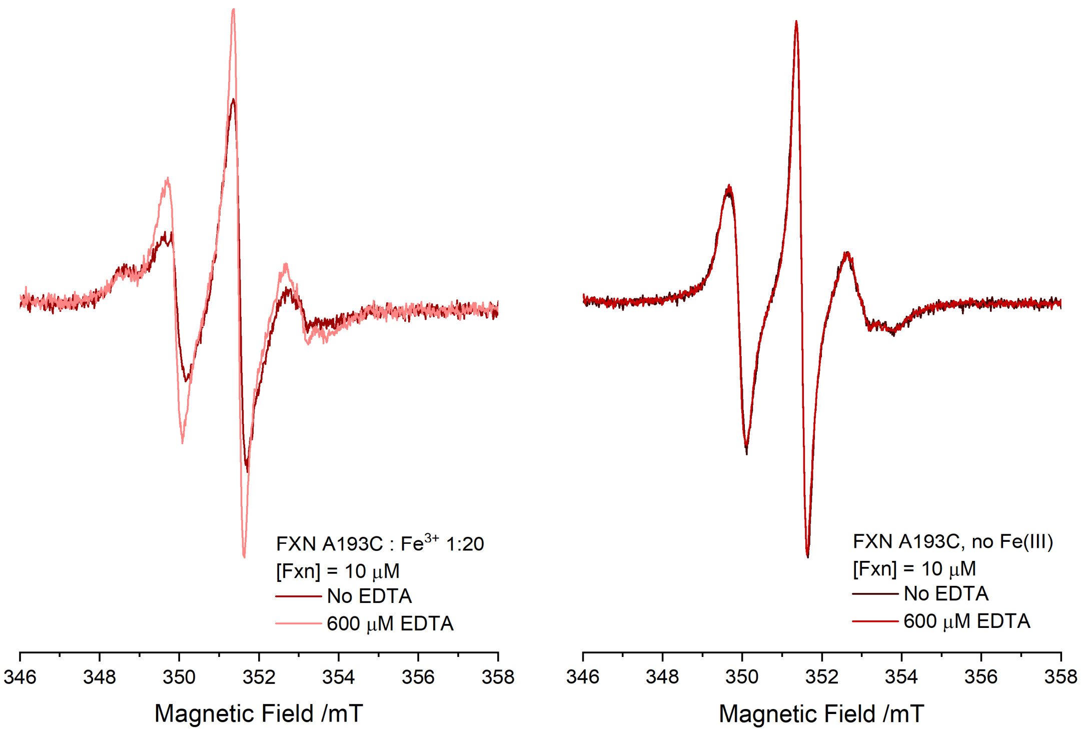

Figure S3 Left, the experimental spectra of A193C in the absence (red) and in the presence (pink) of EDTA. FXN:Fe^{3+} 1:50 ratio, [FXN] = 10 μM. Right, experimental of A193C with no Fe^{3+} present in the absence (red) and in the presence (pink) of EDTA.

M-TETPO-labelled FXN with Fe^{3+}

To check that the protein labelled with M-TETPO behaves like the one labelled with MTSSL, we analyzed three mutants A114C, A114C/D122Y, and A193C in the presence of Fe^{3+}. As can be seen from the spectra reported in FigureS4, all mutants show a progressive immobilization upon increasing Fe^{3+} additions, like their MTSSL-labelled counterparts.

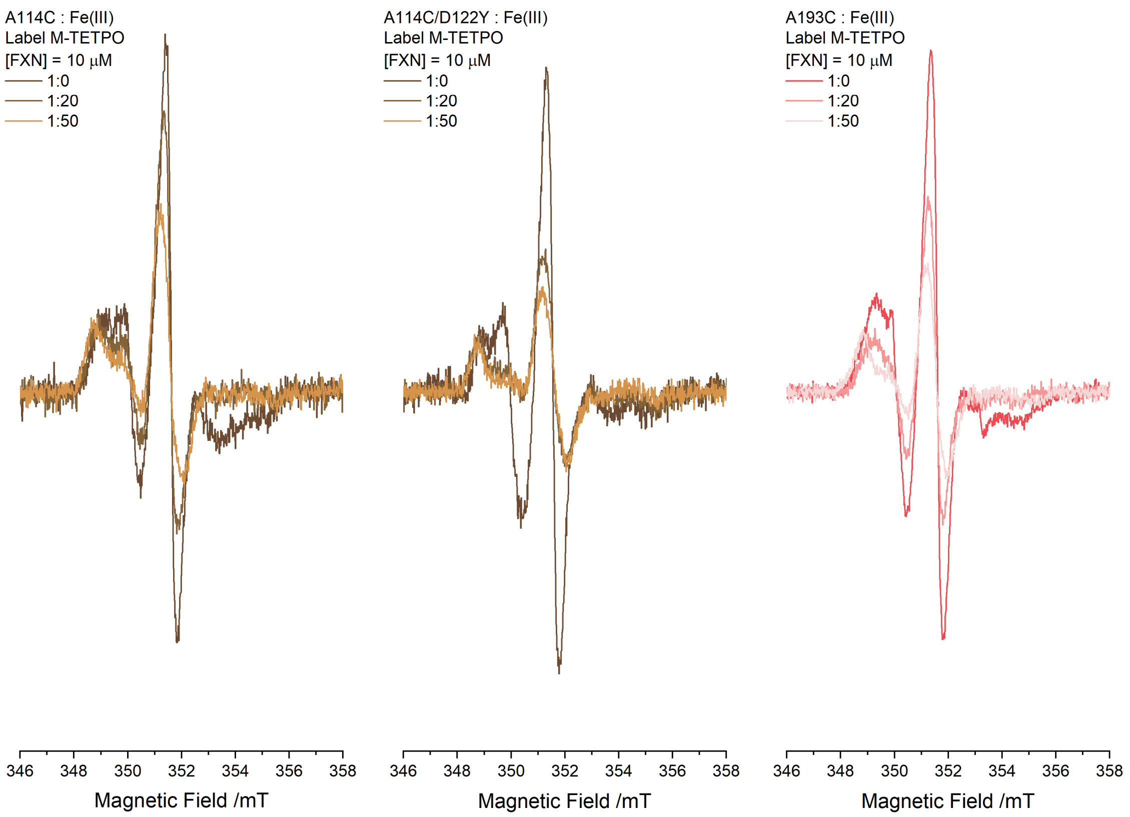

Figure S4 Experimental spectra of A114C (left), A114C/D122Y (middle), and A193C (right) at increasing FXN:Fe^{3+} ratios, [FXN] = 10 μM.

Sidechain conformer analysis of A99C and A114C labelled with M-TETPO

The A99C, A114C, and A114C/D122Y mutants labelled with M-TETPO show a progressive signal loss at increasing amounts of Fe^{2+}, unlike the other labelled positions which show no effects of Fe^{2+}. In the spectra of A114C and A114C/D122Y mutants, the signal loss is accompanied by a change in shape, whereas no change is observed for A99C position. This difference arises from the different conformers of the nitroxide sidechain that are present in the two positions, as shown in the figures below. The conformers have been obtained using the program MMM [Jeschke, G. (2018), MMM: A toolbox for integrative structure modeling. Protein Science, 27: 76-85. doi:10.1002/pro.3269]. As can be seen from FigureS5 top right, A114C has a multitude of possible conformers. The other views show the top three conformers (azure, light blue, and dark blue) that alone account for 76% of the energy accessible conformers. Only the azure and light blue conformers are close to a Fe^{2+} binding site and can thus be reduced, whereas the dark blue one is not. Thus, the reduction in intensity in A114C is accompanied by a change in shape, since the bulky sidechain of M-TETPO does not quickly explore its possible conformations. On the contrary, as can be seen from FigureS6, A99C has only a single conformer that is energetically accessible, all other possible conformations of the sidechain are not present due to steric clashes with the protein backbone. Therefore, when a nearby Fe^{2+} atom reduces the M-TETPO nitroxide only a reduction of intensity can be observed.

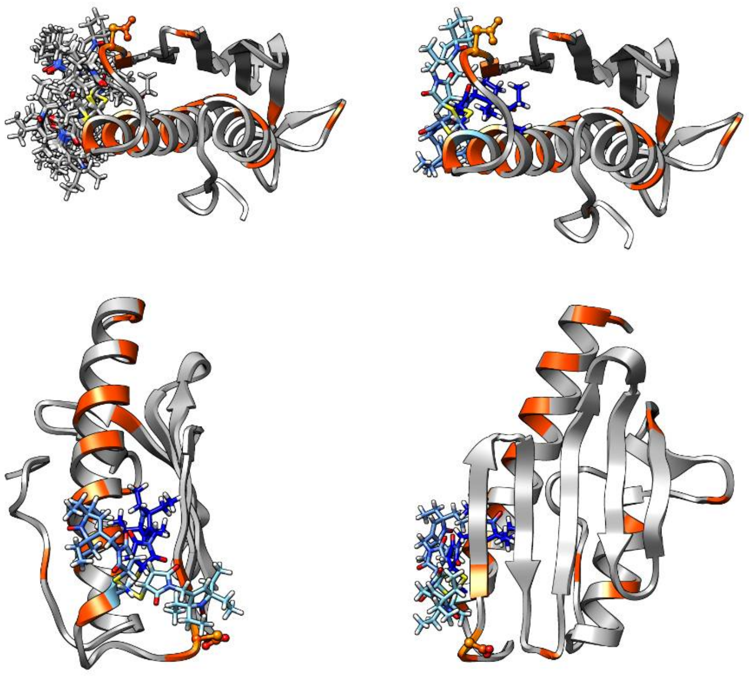

Figure S5 Conformers of the M-TETPO nitroxide attached to the A114 position. Top, view of FXN (PDB ID: 1EKG), with all possible conformers (left) or just the top three (right). Bottom, FXN with the top three conformers shown from different angles.

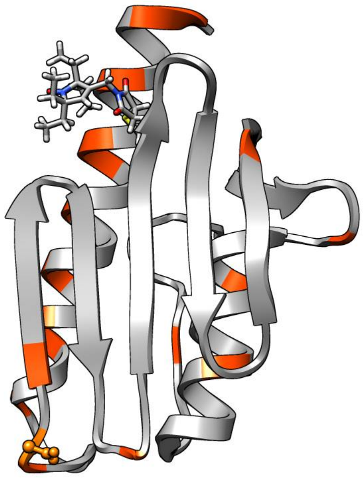

Figure S6 The single sterically allowed conformer of the M-TETPO nitroxide attached to the A99 position of FXN (PDB ID: 1EKG).

Removal of bound Fe^{2+} via chelation by PHEN

We verified the presence of an irreversible redox reaction of the M-TETPO nitroxide with Fe^{2+}, as observed for MTSSL, via chelation of the protein-bound Fe^{2+} by treatment with 1,10-phenanthroline (PHEN). FigureS7 left, shows that, upon PHEN addition to a sample of the A114C/D122Y mutant - 1:50 FXN:Fe^{2+} molar ratio, the spectral intensity does not recover, even though Fe^{2+} has been chelated as verified by the solution turning red. FigureS7 right, shows the quantitative formation of the band, $\varepsilon = 11200\ (cm{\cdot}M)^{-1}$ [*Inorg. Chem.* 1992, 31, 4, 555–559 https://doi.org/10.1021/ic00030a006].

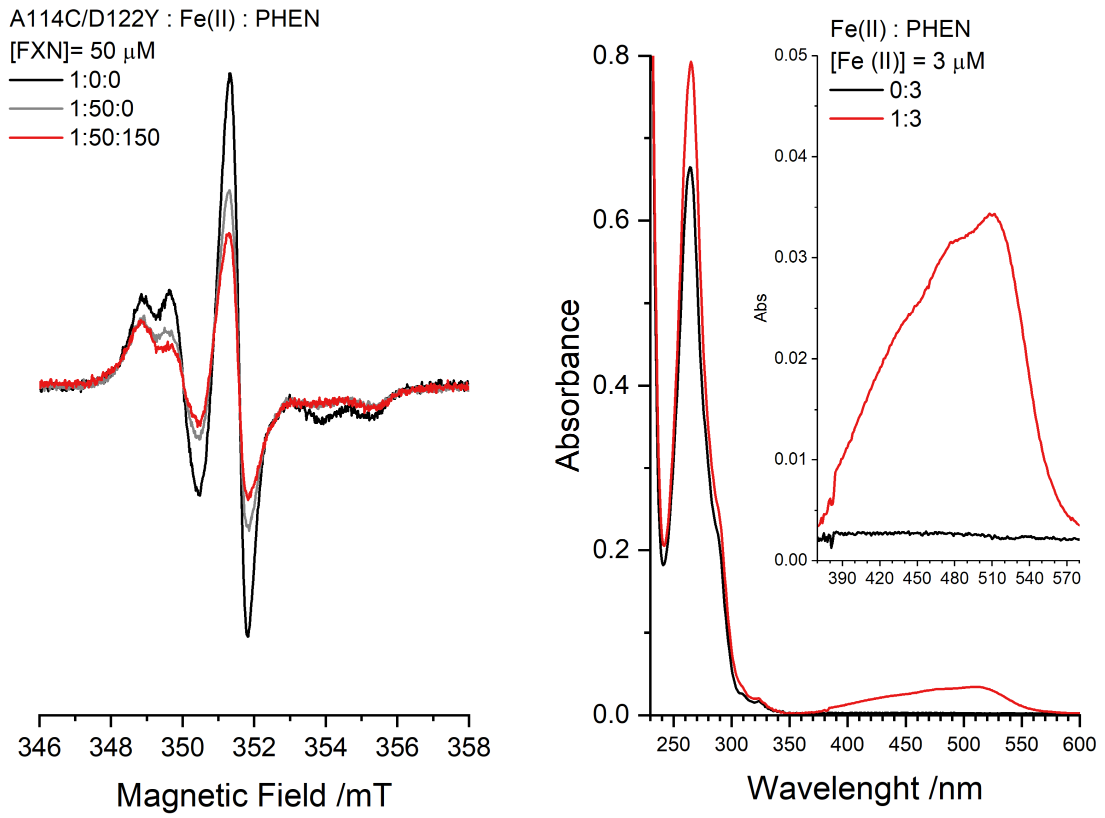

Figure S7 Left, the experimental spectra of A1114C/D122Y alone (black), at a FXN: Fe^{2+} 1:50 ratio (grey), and at a FXN: Fe^{2+}:PHEN 1:50:150 ratio (red); [FXN] = 50 μM. Right, UV-Vis spectra of the solution without (black) and with (red) Fe^{2+}.

Normalized fluorescence spectra

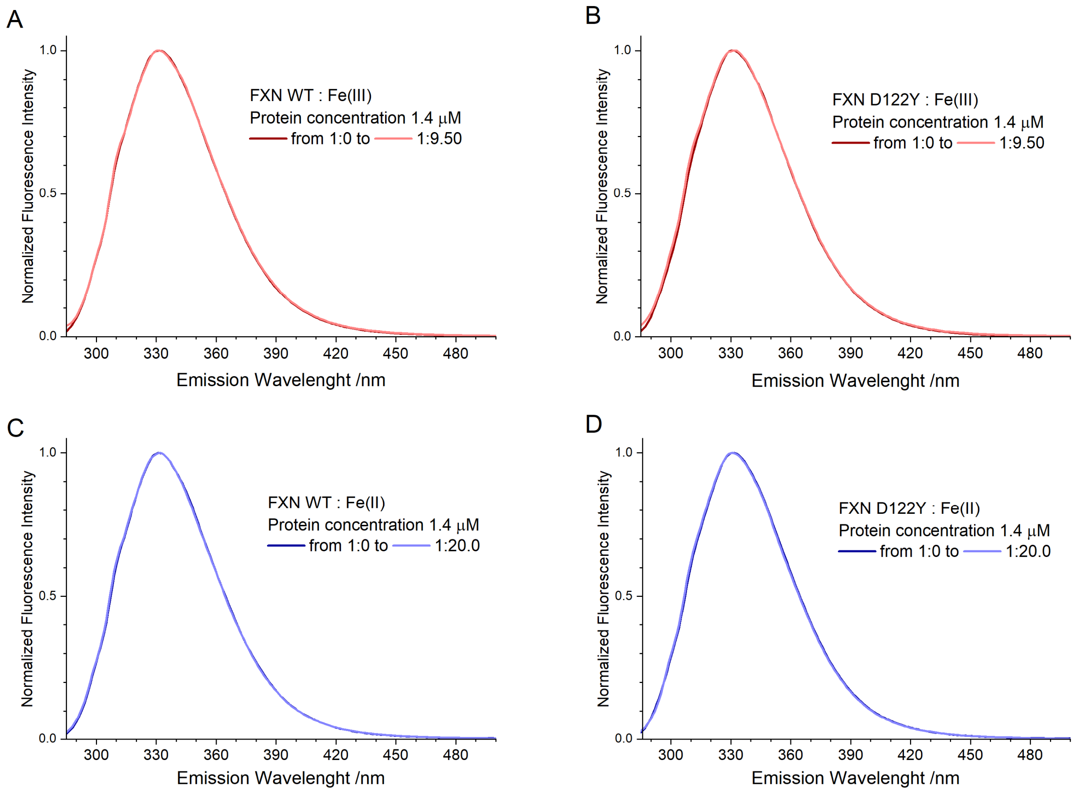

Figure S8 Normalized fluorescence spectra of WT (left, A, C) and D122Y FXN (right B, D): top with Fe(III); bottom with Fe(II).

Fe^{2+} fluorescence quenching analysis

The fluorescence quenching curves of WT and D122Y FXN with Fe^{2+} were analyzed according to the method reported in the literature (Sawyer, W. H.; Winzor, D. J. Theoretical Aspects of the Quantitative Characterization of Ligand Binding. *Curr. Protoc. Protein Sci.* **1999**, *16* (1), 1–40. https://doi.org/10.1002/0471140864.psa05as16). The analysis is reported in Figure S9. Briefly, from the fractional saturation ($fa = \frac{(F-Ff)}{(Fb-Ff)}$, where F is the fluorescence at a given Fe^{2+} concentration, Ff the one without Fe^{2+} and Fb the one at saturating Fe^{2+} concentrations) the number of binding sites on the acceptor (p) is determined at the crossing of the two blue lines as shown in the top part of Figure S9; the result is p=1 for both proteins.

Once the stoichiometry has been determined, the binding function (r) can be evaluated from a titration in which the acceptor concentration is close to the dissociation constant for the binding equilibrium. The value of the dissociation constant Kd is obtained from a linear regression of the titration according to the equation *A.5A.10* in the reference: $\frac{1}{r} = p^{-1} + Kd\frac{1}{pCs}$. The value of r is established at each value of Cs, the concentration of free ligand: $Cs = \overline{Cs} - r\overline{Ca}$, where $\overline{Cs}$ and $\overline{Ca}$ are the total concentration of Fe^{2+} and acceptor (i.e. the protein), respectively.

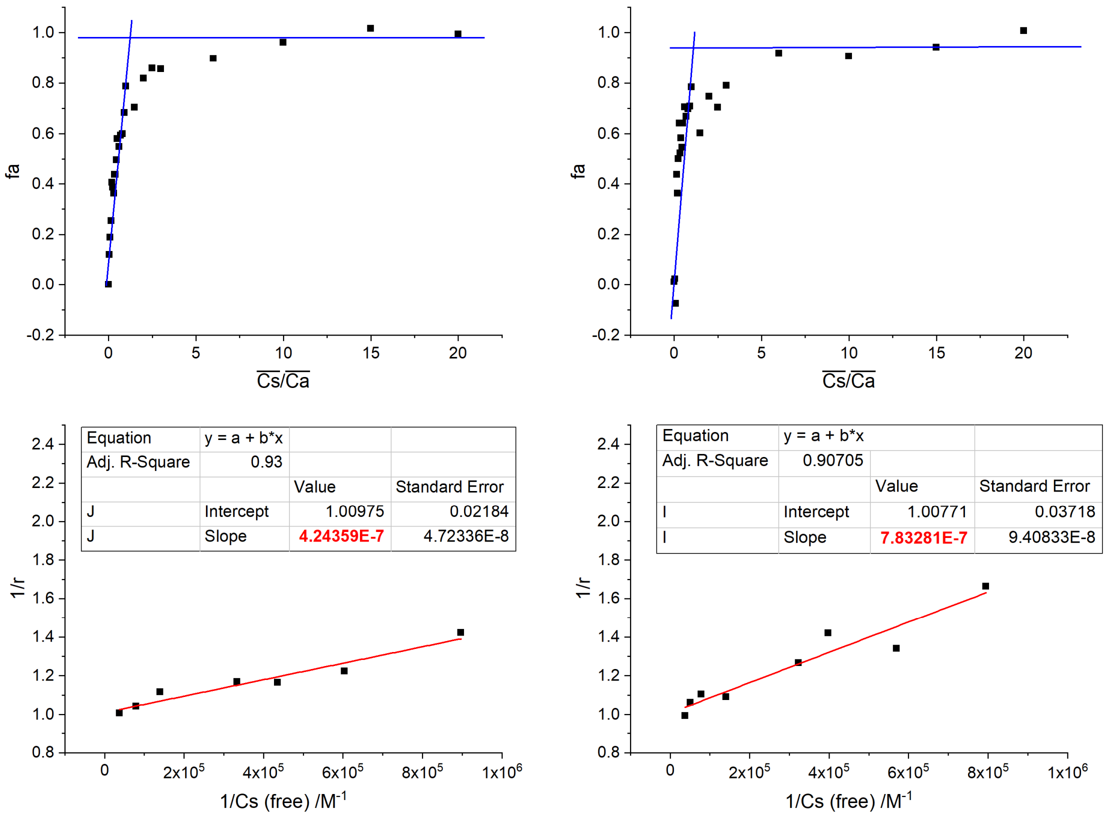

Figure S9 Analysis of the fluorescence quenching experiments with Fe^{2+}, for FXN WT (left) and its D122Y variant (right).

Synthesis of M-TETPO

Chemicals were purchased from Sigma-Aldrich or TCI and were used as received. All experiments were performed under anhydrous conditions and an inert atmosphere of argon and, except where stated, using dried apparatus and employing standard techniques for handling air-sensitive materials. High-resolution mass spectra (HRMS) were performed on a SYNAPT G2 HDMS (Waters) spectrometer equipped with atmospheric pressure ionization source (API) pneumatically assisted. Samples were ionized by positive electrospray mode as follows: electrospray tension (ISV): 2800 V; opening tension (OR): 20 V; nebulization gas pressure (nitrogen): 800 L/h. Analytical thin layer chromatographies (TLC) were carried out on Macherey Nagel DC-Fertigfolien alugram Xtra SIL G plates. Flash column chromatographies were carried out on Merck Kieselgel 60 (230–400 mesh). For EPR measurements to test the products, samples with 0.5 mM concentration of nitroxide were prepared in non-degassed solvents. Experiments were performed on an EMX Bruker machine.

Procedure to prepare M-TETPO from alcohol **1**:

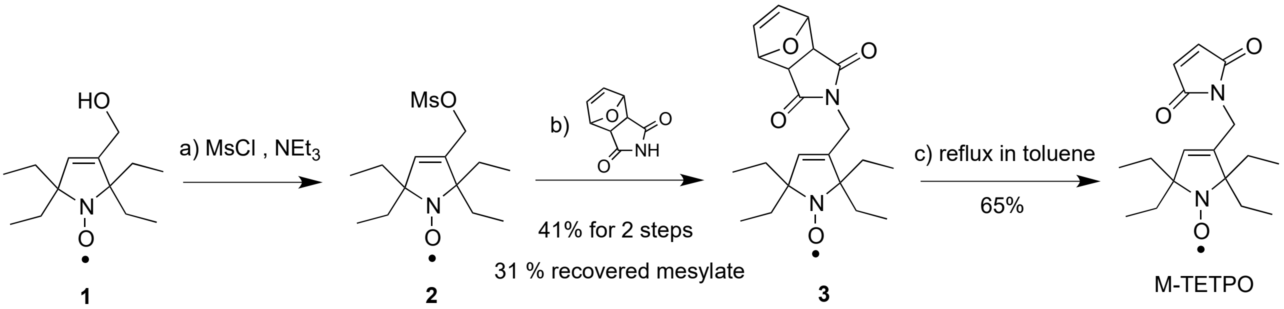

Methanesulfonyl chloride (50 µL, 0.65 mmol) was added to a solution of alcohol **1** (134 mg, 0.59 mmol) and NEt_3 (90 µL, 0.65 mmol) in CH_2Cl_2 (10 mL) at 0°C and the solution was stirred at ambient temperature. After 2h, the reaction was quenched with 1M HCl solution (5 mL) and extracted with CH_2Cl_2 (3 x 10 mL). The combined organic layers were washed with brine (10 mL), dried over $MgSO_4$, filtered and the solvent was removed in vacuum. The residue oil was dissolved in DMF (10 mL), K_2CO_3 (245 mg, 1.77 mmol) and protected maleimide (195 mg, 1.18 mmol) were added. After stirring at 60 °C for 16 h, the reaction was diluted with H_2O (10 mL) and extracted with EtOAc (3 x 10 mL). The combined organic layers were washed with brine (2 x 10 mL), dried over $MgSO_4$, filtered and the solvent was removed in vacuum. The residue was purified by column chromatography (SiO_2, petroleum ether-EtOAc 2:1) to yield unreacted mesylate **2** (55 mg, 0.18 mmol, 31 %, R_f = 0.54) and the compound **3** (90 mg, 0.24 mmol, 41%, R_f = 0.26) as a yellow solid; mp = 118°C. EPR: *g*-value = 2.004; a_N = 14.46 G. IR (ATR): 2967 (w), 2937 (w), 2881 (w), 1703 (s), 1670 (s), 1383 (m), 1088 (m), 880 (m) cm^{-1}. HRMS (ESI-TOF): m/z calcd for $C_{21}H_{29}N_2O_4Na$ [M + Na^+]: 396.2020; found 396.2018.

A solution of compound **3** (74 mg, 0.20 mmol) in toluene (10 mL) was heated to reflux for 3 h. After removal of the solvent in vacuum, the residue was purified by column chromatography (SiO_2, petroleum ether-EtOAc 2:1, Rf = 0.50) to yield M-TETPO (41 mg, 0.13 mmol, 65%) as an orange oil. EPR: *g*-value = 2.005; a_N =

14.36 G. IR (ATR): 2967 (w), 2937 (w), 2880 (w), 1707 (s), 1405 (m), 1152 (m), 828 (m), 693 (m) cm^{-1}. HRMS (ESI-TOF): m/z calcd for $C_{17}H_{25}N_2O_3Na$ [M + Na^+]: 328.1757; found 328.1757.
